# Diagnostic performance of susceptibility-weighted magnetic resonance imaging for the detection of calcifications: A systematic review and meta-analysis

**DOI:** 10.1038/s41598-017-15860-1

**Published:** 2017-11-14

**Authors:** Lisa C. Adams, Keno Bressem, Sarah Maria Böker, Yi-Na Yvonne Bender, Dominik Nörenberg, Bernd Hamm, Marcus R. Makowski

**Affiliations:** Department of Radiology, Charité, Charitéplatz 1, 10117 Berlin, Germany

## Abstract

Since its introduction, susceptibility-weighted-magnetic resonance imaging (SW-MRI) has shown the potential to overcome the insensitivity of MRI to calcification. Previous studies reporting the diagnostic performance of SW-MRI and magnetic resonance imaging (MRI) for the detection of calcifications are inconsistent and based on single-institution designs. To our knowledge, this is the first meta-analysis on SW-MRI, determining the potential of SW-MRI to detect calcifications. Two independent investigators searched MEDLINE, EMBASE and Web of Science for eligible diagnostic accuracy studies, which were published until March 24, 2017 and investigated the accuracy of SW-MRI to detect calcifications, using computed tomography (CT) as a reference. The QUADAS-2 tool was used to assess study quality and methods for analysis were based on PRISMA. A bivariate diagnostic random-effects model was applied to obtain pooled sensitivities and specificities. Out of the 4629 studies retrieved by systematic literature search, 12 clinical studies with 962 patients and a total of 1,032 calcifications were included. Pooled sensitivity was 86.5% (95%-confidence interval (CI): 73.6–93.7%) for SW-MRI and 36.7% (95%–CI:29.2–44.8%) for standard MRI. Pooled specificities of SW-MRI (90.8%; 95%–CI:81.0–95.8%) and standard MRI (94.2; 95%–CI:88.9–96.7%) were comparable. Results of the present meta-analysis suggest, that SW-MRI is a reliable method for detecting calcifications in soft tissues.

## Introduction

Calcifications can be prognostic markers in a variety of locations or tissues^1–3^. CT is currently used as the reference standard for the detection of calcifications. However, it is associated with the disadvantage of exposing patients to ionizing radiation. Standard MRI, on the other hand, is radiation-free, but insensitive to calcifications^4^.

SW-MRI, which is gradually being adapted into clinical practice, has shown the potential to overcome this drawback of MRI. It is a high spatial resolution MRI gradient echo (GRE) technique, relying on susceptibility differences between tissues to produce a contrast different from T1, T2 and T2* weighted images^5^. While most clinically used MR sequences mainly use the information derived from magnitude images and discard the phase information^6^, the development of phase filters has enabled the removal of artefacts and the generation of a tissue contrast based on local susceptibility differences. The filtered phase image is useful to differentiate between diamagnetic (e.g. calcium) and paramagnetic (e.g. blood products, iron) compounds and to identify calcifications^5,6^. In the years since its introduction, it has been suggested that SW-MRI can offer additional diagnostic information across a wide spectrum of pathologies without radiation exposure^7^.

Previous studies reporting the diagnostic performance of SW-MRI and MRI for the detection of calcifications are partly inconsistent and based on single-institution designs. To date, there has been no systematic and qualitative synthesis of this evidence.

Therefore, we sought to compare and determine the potential of SW-MRI for detecting calcifications in brain and body soft tissues. More specifically, we proposed the following explicit statement of questions (PICO): Patients with intracranial or extracranial calcifications; Index test of SW-MRI used to identify calcifications and differentiate it from other tissues; if direct Comparison to standard MRI was performed in the same patients, this data will also be extracted and used for comparison; Outcome (presence or absence of calcifications) was assessed based on the reference standard CT.

## Results

**Figure 1:**
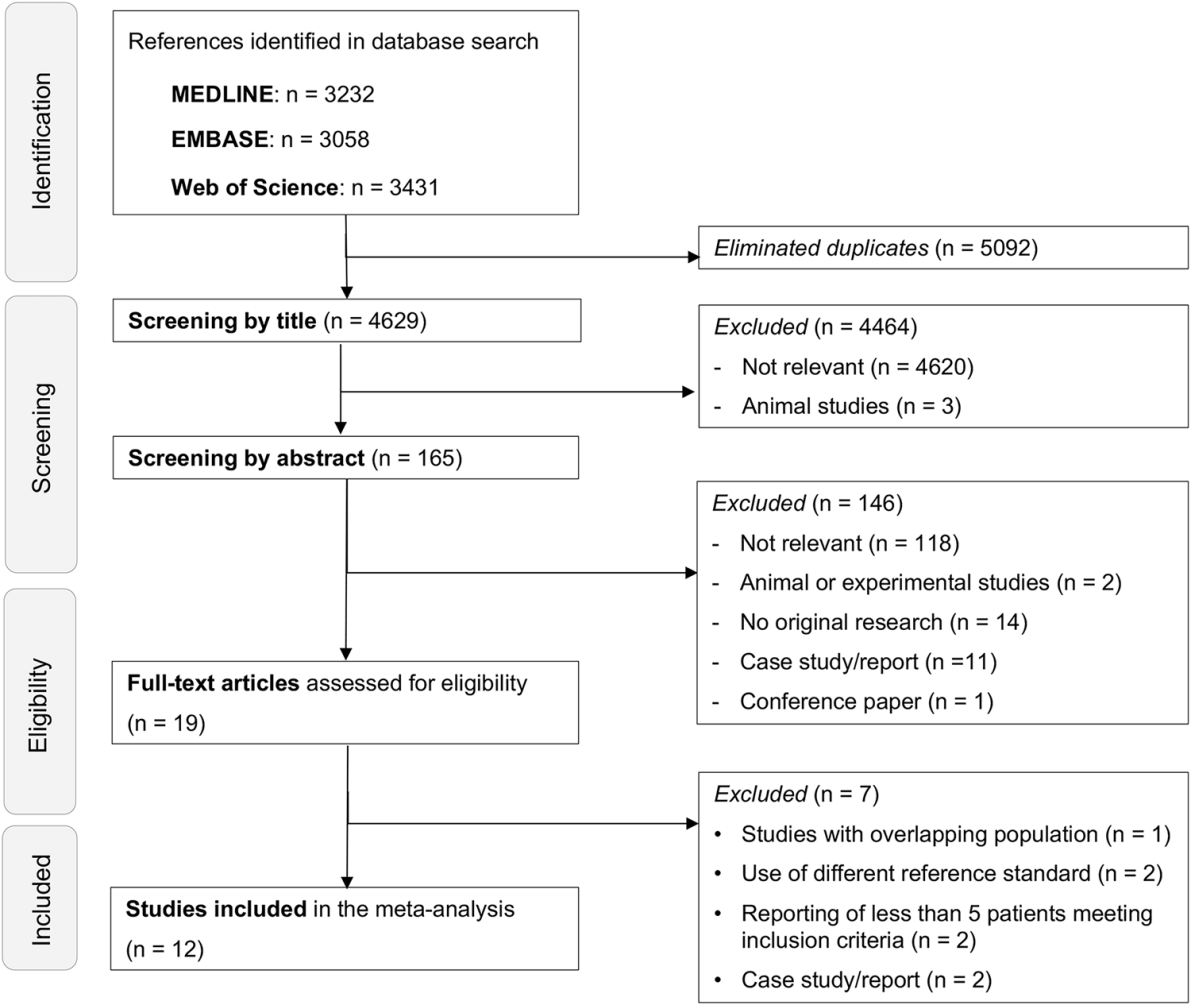
Study selection flowchart.

A Preferred Reporting Items for Systematic Reviews and Meta-Analyses (PRISMA) flowchart^8^ was used to report the decisions made in the course of the study selection process (see Supplementary Information S1 and see Fig. 1 for the study selection flowchart). Overall, 12 suitable clinical studies were identified and included in the present meta-analysis^1,2,9–18^. See Supplementary Information S2 for a list of studies, which were excluded at the level of full-text search, including the individual reason for exclusion.

### Patient and study characteristics

In total, 962 patients (male = 554, female = 257, sex unknown = 151) were included with an average age of 52.2 years. MRI and CT examinations were performed within a period of 0.5 to 90 days. All the studies were single-centre and study design was described as prospective in six of the studies, as retrospective in five of the studies and remained unclear in one case. Six of the studies were performed on 1.5T scanners, five of the studies at 3T and one of the studies was performed at both 1.5 and 3T. The characteristics of the patients and studies included in this meta-analysis are provided in Table 1.

**Table 1:** Study Characteristics of the Included Studies.

Source Study	Year	Case Date Range	Study Design	Sample Size	Region	Characteristics of imaging units					Demographic data		
						MRI			CT				
						Interval CT/MRI (days)	Field strength	Image Unit Manufacturer	Detector rows	Attenuation threshold (HU)	Men	Age (years)	Age range (years)
Adams *et al*.	2017	01/2014–10/2016	retrospective	346	Germany	<90	1.5	Siemens	64, 320	130	173	58.7	18–95
Bai *et al*.	2013	06/ 2011–09/2012	prospective	76	China	N/A	3	Siemens	16	100	76	69.5	49–91
Barbosa *et al*.	2015	N/A	retrospective	6	Brazil	90 (1 with 300)	3	Philips	16	N/A	4	N/A	41–54
Berberat *et al*.	2014	N/A	retrospective	11	Switzerland	N/A	1.5	Siemens	320	N/A		58.9	39–74
Chen *et al*.	2014	03/2010–08/2010	prospective	38	China	0.5–5	3	GE Healthcare	16	100	24	33	6–75
Dou *et al*.	2016	06/2011–10/2014	prospective	156	China	N/A	3	N/A	16	100	156	67.2	56–83
Gronemeyer *et al*.	1992	N/A	unclear	19	USA	N/A	1, 1.5	Siemens	N/A	200	N/A	N/A	N/A
Saini *et al*.	2009	2006–2009	retrospective	137	India	N/A	1.5	Siemens	N/A	N/A	16	22.6	5–32
Yamada *et al*.	1996	N/A	prospective	49	Japan	30	1.5	Siemens	N/A	N/A	N/A	N/A	N/A
Yang *et al*.	2009	N/A	prospective	18	China	N/A	3	Siemens	64	130	11	65	39–79
Zhu *et al*.	2008	09/2007–12/2007	prospective	35	China	<1	1.5	GE Healthcare	16	90	19	39	09–65
Zulfiqar *et al*.	2012	2001–2010	retrospective	71	USA	N/A	1.5, 3	GE, Philips, Siemens	N/A		33	42.5	14.5–78.5

Note. – N/A = data not available.

### Quality assessment

**Figure 2:**
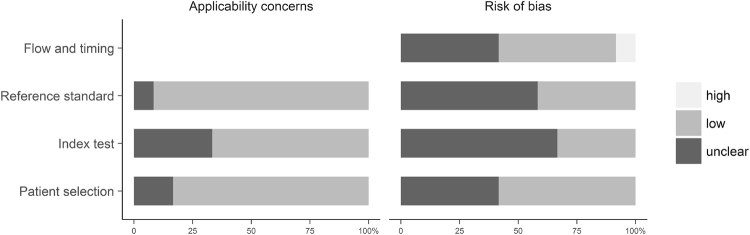
QUADAS-2 assessment of study quality. Grouped bar charts of the QUADAS-2 scores are expressed as percentage of the 12 included studies meeting each criterion. For each domain, the proportion of the included studies that indicate low/high/unclear concerns of applicability or risk of bias are displayed in light grey = “yes” answers (good quality), dark grey bar = “unclear” answers, and medium bar = “no” answers (low quality).

The detailed results of the QUADAS-2 (QUality Assessment of Diagnostic Accuracy Studies) evaluation of the methodologic quality (risk of bias and concerns regarding applicability) are presented in Fig. 2. The majority of the studies were assessed as having low concerns regarding applicability. Methods of reconstruction and especially the interpretation of the CT and MRI scans were poorly described. In five of the studies the risk of bias for patient selection was unclear due to incomplete reporting on the methods of patient selection. Risk of bias related to the conduction of the index test or the reference standard was unclear in eight or seven of the studies, as no information was provided on whether the radiologists were blinded to the reference standard or the index test. Several studies did not describe, if there was an appropriate interval between the interpretation of the index test(s) and the reference standard^10,14,15,17,19,20^. The time intervals given ranged from less than 24 hours to three months. For one patient, the interval between the MRI and the CT examination was 10 months, because of which the corresponding study was rated as having a high risk of bias related to flow and timing.

### Assessment of calcifications

**Figure 3:**
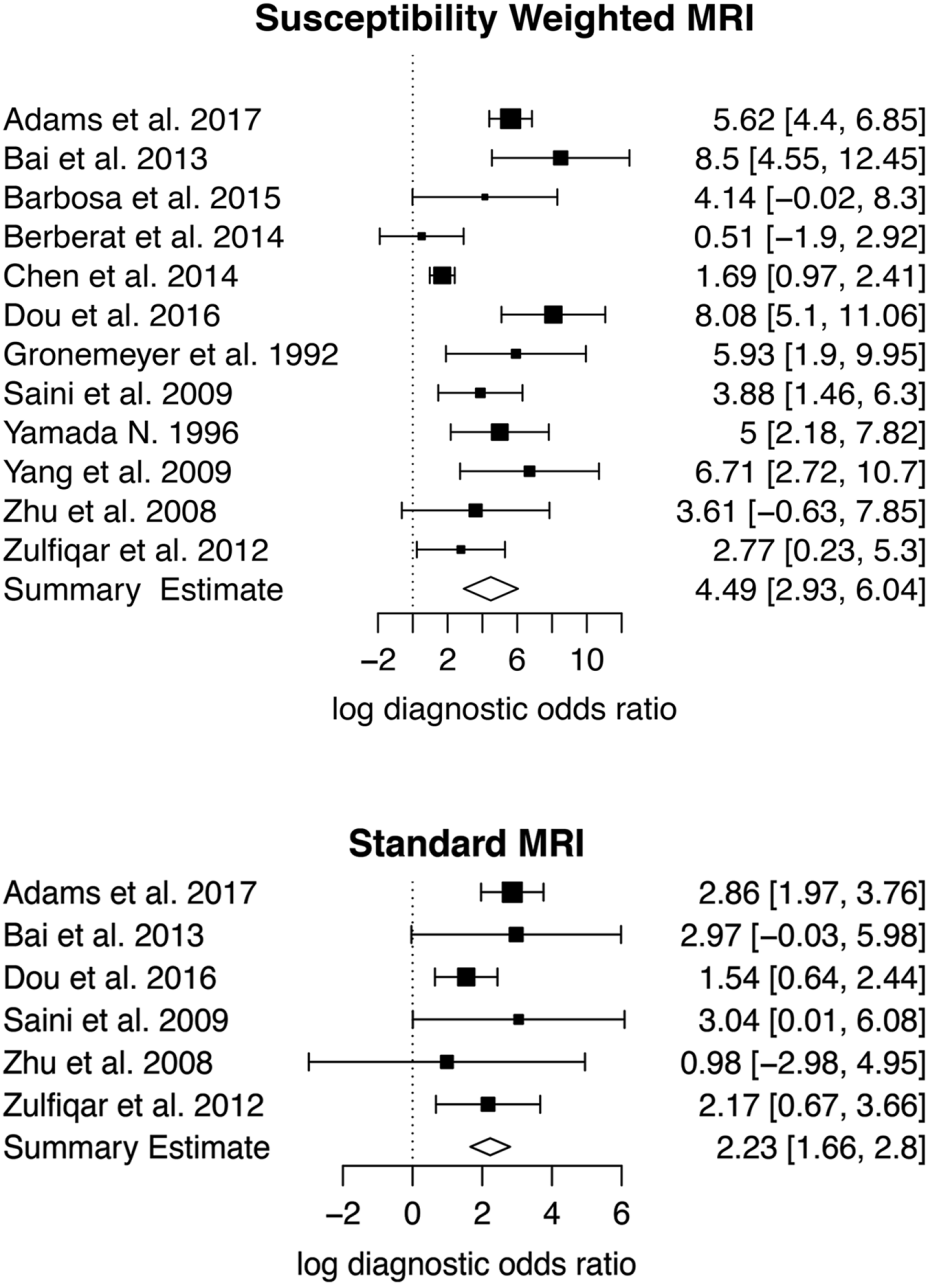
Forest plots showing the log diagnostic odds ratios (black squares) for susceptibility weighted imaging and standard magnetic resonance imaging (MRI) (where available) of each study with 95% confidence intervals (horizontal lines). The area of each square is proportional to the study’s weight in the meta-analysis and the summary measure of effect is plotted below as a diamond. An effect size of zero is indicated by the vertical dashed line.

**Figure 4:**
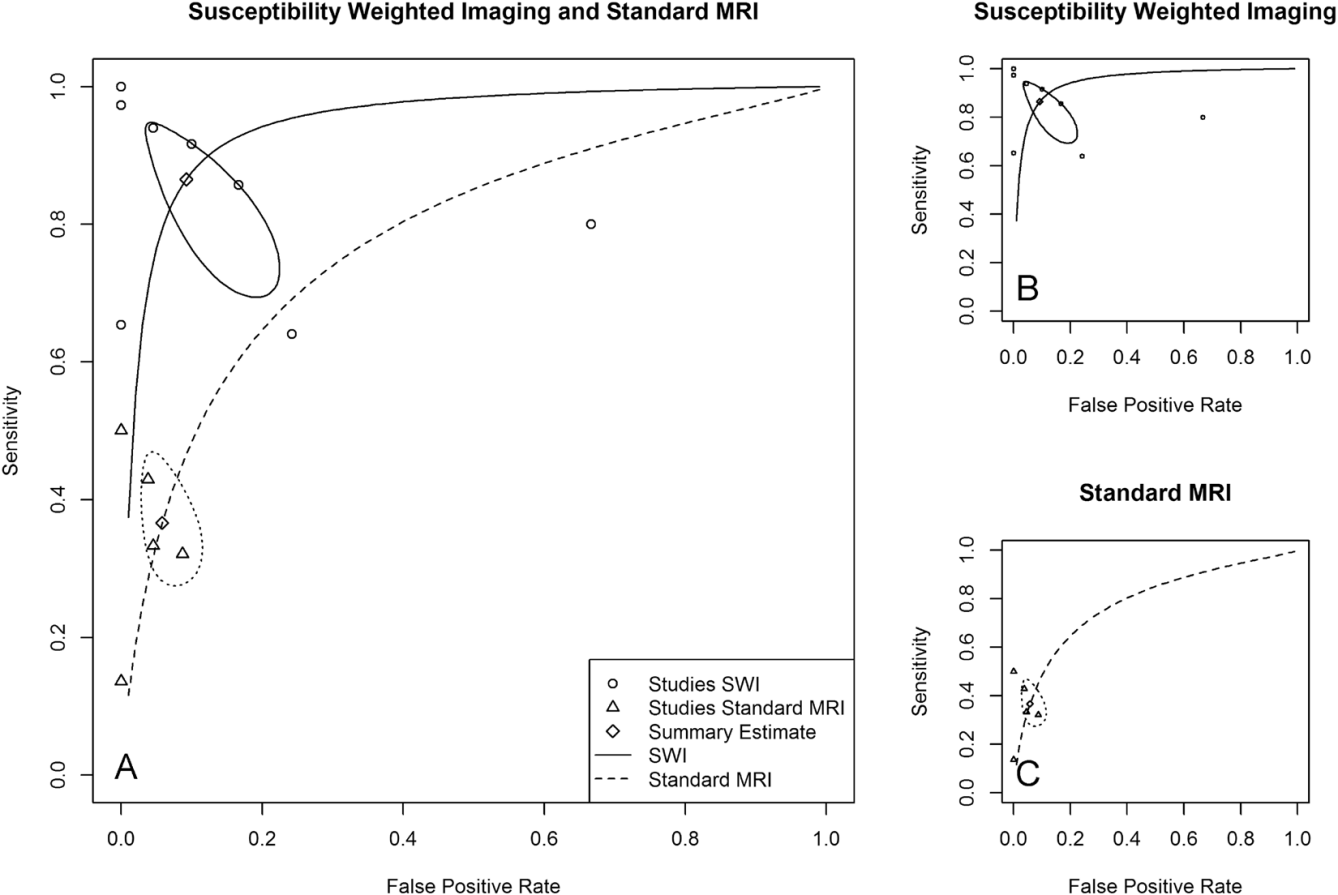
Summary ROC curves of overall diagnostic accuracy for susceptibility weighted magnetic resonance imaging (SW-MRI) and magnetic resonance imaging (MRI) (**A**), for the overall diagnostic accuracy of SW-MRI (**B**) and the overall diagnostic accuracy of MRI (**C**). The false positive rate (1-specificity) is plotted against the false positive rate (1-specificity). The sROC curves show a deviation of the data points and especially for standard MRI, the extrapolation of the sROC curve is highly vulnerable to outliners.

The diagnostic performance of SW-MRI for the identification of calcifications was analysed in all studies. The resulting forest plots of the log diagnostic odds ratios are given in Fig. 3. Table 2 provides an overview of the detailed results for sensitivities and specificities. The pooled sensitivity for SW-MRI was 86.5% (95% CI: 73.6–93.7%) and the pooled specificity was 90.8% (95% CI: 81.0–95.8%). sROC curves of overall diagnostic accuracy for SW-MRI and MRI are provided in Fig. 4. The area under the curve (AUC) for SW-MRI was 0.95 (MRI: 0.78). Especially for standard MRI, the extrapolation of the sROC curve is highly vulnerable to outliers.

**Table 2:** Overview of the True Positives, True Negatives, False Positives and False Negatives for SW-MRI and MRI.

Source Study	Sample Size	Number of Cases	SW-MRI				MRI			
			True Positives	False Negatives	True Negatives	False Positives	True Positives	False Negatives	True Negatives	False Positives
Adams *et al*.	346	346	203	11	127	5	92	122	127	5
Bai *et al*.	76	76	22	0	54	0	3	19	54	0
Barbosa *et al*.	6	6	4	0	2	0	N/A	N/A	N/A	N/A
Berberat *et al*.	11	11	4	1	2	4	N/A	N/A	N/A	N/A
Chen *et al*.	38	151	57	32	47	15	N/A	N/A	N/A	N/A
Dou *et al*.	156	163	109	3	51	0	36	76	63	6
Gronemeyer *et al*.	19	19	12	0	7	0	N/A	N/A	N/A	N/A
Saini *et al*.	137	22	11	1	9	1	6	6	10	0
Yamada *et al*.	49	140	66	35	39	0	N/A	N/A	N/A	N/A
Yang *et al*.	18	29	19	0	10	0	N/A	N/A	N/A	N/A
Zhu *et al*.	35	56	55	1	N/A	N/A	41	15	N/A	N/A
Zulfiqar *et al*.	71	13	6	1	5	1	9	18	42	2

Notes. – N/A = data not available, SW-MRI = Susceptibility weighted magnetic resonance imaging.

In half of the studies^1,2,9,15,17,21^ direct comparison to standard MRI was performed with a pooled sensitivity of 36.7% (95% CI: 29.2–44.8%) and a pooled specificity of 94.2% (95% CI: 88.9–96.7%).

The marginally lower specificity of SW-MRI compared to standard MRI is most likely to be explained by the inverse proportional relationship of sensitivity and specificity.

To evaluate, if the differences observed between standard MRI and SW-MRI were significant, an analysis of variance (ANOVA) was performed. Sensitivity and specificity differed significantly for MRI and SW-MRI, when the imaging method was added as a covariate (p < 0.0001).

The data retrievable from Zhu *et al*.^22^ only permitted the calculation of the sensitivity for the detection of calcifications and could thus not be included into the bivariate model.

In the study by Chen *et al*.^12^, SW-MRI was compared against quantitative susceptibility mapping (QSM), whereby SW-MRI showed a significantly lower diagnostic performance.

### Heterogeneity and publication bias

The Chi-squared test suggested heterogeneous results for SW-MRI (p < 0.001 for sensitivity and for specificity) and also partly for MRI (p < 0.001 for sensitivity; p = 0.11 for specificity). Covariate analysis could only explain part of the observed heterogeneity. Adding the location of the lesion (intracranial/body) as a covariate to the model showed the greatest effect on the variability estimates for SW-MRI, whereby adding “intracranial” as a covariate decreased variability estimates from 1.12 and 1.09 (for sensitivity and specificity) to 1.04 and 0.89.

Considering the relatively small number of studies included in the present meta-analysis (n = 12), funnel plots and regression tests of asymmetry based on it may be inconclusive as a tool for detecting publication bias^23^. Although the regression test of asymmetry revealed a negative test result (p = 0.45 for SW-MRI and p = 0.19 for standard MRI), this cannot necessarily be taken as indicator of a low probability of publication bias. Especially with regard to standard MRI, with only six studies evaluating the performance of both SW-MRI and standard MRI, publication bias cannot be excluded.

## Discussion

Major advances in MRI have led to the recent development of SW-MRI, which has opened the door to an improved non-invasive detection of even small amounts of calcification and haemorrhage. Over the last decade, several literature reviews on SW-MRI have been published^5,24–32^, but to date there has been no systematic approach, critically evaluating and combining the results of comparable studies.

To our knowledge, this is the first meta-analysis to focus on the diagnostic performance of SW-MRI for the detection of calcifications. Pooled sensitivity and specificity estimates for SW-MRI were high. In the studies, that evaluated the performance of both standard MRI and SW-MRI, above two times more patients could be correctly assessed with SW-MRI compared to standard MRI.

Traditionally, CT is considered the reference standard for the detection of calcifications. However, it is associated with radiation exposure and accounts for the majority of the radiation exposure related to medical imaging^33^. This is especially relevant in children and younger patients, who are at a higher risk of developing radiation-induced tumors, infertility and other side effects, and in patients facing multiple follow-up examinations. Therefore, the reduction of radiation dose has become a major concern in clinical routine.

In this context, SW-MRI can offer an alternative radiation-free approach. Its development began in the 1990s with the introduction of “phase imaging” as a means to map susceptibility. After the acquisition of the magnitude and phase images, raw phase images are unwrapped and further processed, usually by transformation into a phase mask, which is then multiplied by the magnitude image^34^. Advances in phase unwrapping and background phase removal have been among the key steps to reduce artifacts and enhance tissue phase contrast. Over the years, various unwrapping and post-processing techniques such as Fourier-based unwrapping, Homodyne-filtering, Gaussian filtering and phase-unwrapping high-pass filtering have been developed in order to reduce artifacts and to enhance the susceptibility contrast^6,34^. So far, there have only been a limited number of studies comparing the performance of the different post-processing approaches and the influence of different filter types, but a recent study suggested, that phase wrapping followed by high-pass filtering might perform most accurately^34^.

The fields of clinical application for SW-MRI include the imaging of venous blood in acute or chronic ischemia, the visualization of the vascularization, haemorrhage and calcification of tumors, the identification of epilepsy-associated calcified and vascular abnormalities and measuring calcification or iron deposition in neurodegenerative diseases^2,9–12,14,15,18,22,35–39^. It has been indicated that with regard to the differentiation between small calcification and haemorrhage, SW-MRI might even be superior to the reference standard CT, as due to a considerable overlap of attenuation values these conditions are not always easy to distinguish on CT scans^19,40^.

Besides brain imaging, possible clinical applications of SW-MRI have also been extended to other areas, among which belong the detection of prostatic calcification^1,21^, the identification of calcific tendonitis^41^ and subacromial spurs^42^ as well as the visualization of peripheral vessel calcifications^16^.

Depending on their location and pattern, calcifications can hint at various pathologies. In the brain, calcification is a very important factor in the diagnosis of brain neoplasms. As different tumors show overlapping features in different diagnostic imaging modalities, detecting whether a tumor is associated with calcifications is useful in narrowing the differential diagnosis. Tumors frequently showing intratumoral calcification include oligodendrogliomas, meningiomas, craniopharyngiomas, pineal gland tumors and ependymomas^2,43^. For prostate cancer, which has become one of the major challenges to public health, the identification of prostatic calcifications and differentiation from haemorrhage is an important diagnostic step, as the reliable detection of haemorrhage can be used as a biomarker for cancerous tissue^44^. Prostatic calcifications can indicate several urological diseases and symptoms such as underlying inflammation^45^. Within rotator cuff tendons, calcium deposition is a diagnostic clue for calcific tendonitis^41^. In vessels, calcifications can be a sign of advanced stages of atherosclerosis^20^. With regard to vessel calcifications and the imaging of complex plaque features with intraplaque haemorrhage and/or inflammation, SW-MRI has advantages over conventional imaging techniques by being able to detect even small foci of haemorrhage and to differentiate them from calcification^32^.

The various possible clinical applications of SW-MRI for detecting calcifications are also displayed by the studies included in the present meta-analysis: Pertaining to the neuroaxis, the aims of the original studies included the general detection and differentiation of intracranial calcifications by susceptibility weighted magnetic resonance imaging (SW-MRI) such as in the studies by Barbosa, Chen, Gronemeyer, Yamada and Zhu *et al*., the specific assessment of pineal gland calcifications (Adams *et al*.), of epilepsy-associated calcifications (Saini *et al*.) and of intratumoral calcifications in intracranial gliomas (Berberat *et al*. and Zulfiqar *et al*.)^9–12,14,15,17,18,46^. Pertaining to the body-axis, Bai and Dou *et al*. aimed to show the diagnostic performance of SW-MRI for the specific detection of prostatic calcifications and Yang *et al*. wished to demonstrate the usefulness of SW-MRI for the assessment of vessel wall calcifications in major peripheral arteries^20,47,48^. The common primary endpoint of all included studies was the detection of calcium-phosphate deposition in brain and body soft tissues.

The present meta-analysis has several limitations. First, the number of studies that met our inclusion criteria was relatively low, whereby the small study size is especially relevant with regard to MRI. Therefore, no subgroup analyses were performed, as the sample size was considered too small to obtain reliable results. Also, the included studies were heterogeneous, e.g. regarding the size of the study populations and the location and type of the calcifications, whereby covariate analysis could only explain part of this heterogeneity. Furthermore, not all authors provided sufficient information about the study design and the assessment of the index text and/or the reference standard. Another aspect is, that possible bias could have resulted from the facts that inclusion criteria were not standardized and that the studies were conducted in different clinical settings. Although CT is considered the best imaging technique for the detection of calcifications, the additional use of histopathology to confirm the diagnosis would have been superior, but was only applied in two of the studies^15,19^. A further limitation is, that covering a large time period, this meta-analysis includes studies with different algorithms and post-processing techniques. While the SW-MRI image contrast is relatively consistent, the quality of SW-MRI images naturally depends on the robustness and accuracy of the post-processing on the phase image^34^. Therefore, the quality of the image data in the present meta-analysis may differ. Also, due to the variance in diagnostic performance and the relatively small number of data points, the extrapolation of the sROC curve is highly vulnerable to outliners, especially for standard MRI; and assumptions on significant differences between standard MRI and SW-MRI cannot be made solely based on the sROC curves. Furthermore, the SW-MRI phase image has the disadvantage of aliasing if the field is large enough so that the phase exceeds π radians, which makes it difficult to obtain the exact shape and extent of especially larger calcifications^4^. Finally, we did not include grey literature, but only published studies, which might cause a selection bias, as potentially unpublished data could have shown unexpected results, because of which it might not have been intended for publication or may not have met the journal’s criteria.

As SW-MRI does not enable quantitative measurements, new susceptibility-based techniques, such as QSM, are currently developed and implemented^49^. QSM has shown the potential for more accurate measurements of total volume and susceptibility and may thus be a solution for quantifying calcification or haemorrhage on MR images^12^. So far, there have only been a limited number of studies published on the diagnostic performance of QSM in the detection of calcifications, which suggested a convincing sensitivity and specificity^12,50–52^. In a comparison study of SW-MRI and QSM, Chen *et al*. showed, that QSM might achieve a higher sensitivity and specificity than SW-MRI (80.5% vs. 71% and 93.5% vs. 76.5%) in the detection of intracranial calcifications. Therefore, QSM may play a significant role in the future applications of SW-MRI and may enable a reliable differentiation and detection of soft tissue calcium deposits in various clinical applications, with initial clinical evidence affirming its effectiveness and its potential superiority to SW-MRI. However, more studies are warranted for confirmation.

In conclusion, this meta-analysis shows that SW-MRI is a reliable technique for the detection of calcifications with an accuracy close to CT. Studies that evaluated the performance of both standard MRI and SW-MRI suggested, that the diagnostic performance of SW-MRI was superior to standard MRI. However, further large, multi-centre and prospective studies are required in order to confirm these findings.

## Materials and Methods

The present meta-analysis is in accordance with the guidelines provided by the PRISMA^8^ (see checklist). The protocol was registered with PROSPERO (International Prospective Register of Systematic Reviews; registration number CRD42017059736). Only studies using SW-MRI for the detection of calcifications with CT as the standard of reference were identified. The literature search was performed using Pubmed (MEDLINE), OvidSP (EMBASE) and Web of Science (ISI). The present meta-analysis is exempt from ethical approval of the Institutional Review Board, as the analysis only involves de-identified data and all the included prospective studies have received local ethics approval.

### Search Strategy and study selection

A comprehensive literature search was performed based on the following combination of MeSH terms and keywords for a PubMed database search: ((SWI[All Fields] AND (“magnetics”[MeSH Terms] OR “magnetics”[All Fields] OR “magnetic”[All Fields])) OR ((“disease susceptibility”[MeSH Terms] OR (“disease”[All Fields] AND “susceptibility”[All Fields]) OR “disease susceptibility”[All Fields] OR “susceptibility”[All Fields]) AND weighted[All Fields]) OR (“quantitative susceptibility”[All Fields] AND “calcification”[All Fields])) OR (“phase image”[All Fields] AND “calcification”[All Fields]). Corresponding keywords were used for OvidSP and Web of Science data search.

All published original study designs in English or German language, that used SW-MRI for the detection of calcifications, were considered eligible. The final search was conducted on March 24, 2017. After the removal of duplicates, the retrieved titles and abstracts were independently scanned by two investigators and, with regard to the inclusion and exclusion criteria, either discarded or retained for further evaluation. Full text versions of relevant studies were extracted for further in depth evaluation. The reference lists of all included studies were checked manually in order to identify other relevant papers. In some studies, only a subset of the patients met the inclusion criteria and only these were included. *Inclusion criteria* were as follows:

(a) Human patients with calcifications.

(b) Use of unenhanced susceptibility-weighted MRI as the index test for the detection of calcifications.

(c) Results of the index test being verified against the reference standard CT.

(d) Data retrievable to calculate a 2 × 2 table (or sufficient data to calculate either sensitivity or specificity).

(e) Reporting of >5 patients meeting the inclusion criteria.

Studies were excluded if they included overlapping samples. If the patient sample data was published in more than one publication, the latest study with the largest patient sample was selected and the duplicate study was removed.

### Data extraction and quality assessment

Data were independently extracted by two authors, by use of standardized data extraction sheets. The extracted data included information on: First author, journal and year of publication, the number of (included) patients, patient age (mean, standard deviation, range), false positives/negatives and true positives/negatives for SW-MRI and MRI (if available), the number of patients excluded (because of study overlap, different index test, no or different reference standard), technical parameters of CT and MRI imaging, absolute attenuation threshold used to differentiate calcification from other tissues in CT. Studies with multiple readers and different numbers of true positives, true negatives, false positives and false negatives were averaged in order to obtain study-level data and, if necessary, rounded to the nearest whole number. All ensuing disagreements were resolved by consensus.

The QUADAS-2 tool was used. It comprises four domains, which are patient selection, index test, reference standard, and flow and timing^53^, which are assessed in terms of risk of bias and concerns regarding applicability. A time span of three months was considered an acceptable interval between MRI and CT imaging, given the slow progression of calcifications. The tool was applied to all studies by two independent investigators. Disagreements were resolved by consensus.

### Statistics and data analysis

Data was exported as comma separated values and further processed using ‘R’ Statistical Software (Version 3.2.2, R Development Core Team, Vienna, Austria, 2016). Data from the 2 × 2 tables was summarized in forest plots for each study. Forest plots of log diagnostic odds ratios were generated along with their 95% confidence intervals (CI). Since some of the 2 × 2 tables included zero cells, a continuity correction of 0.5 was done prior to regression analysis. The bivariate diagnostic random-effects model by Reitsma *et al*.^54^ was used to compare pooled estimates of sensitivity and specificity for the index tests (SW-MRI, standard MRI, if available). Pairs of sensitivity and specificity are analysed together, whereby any correlation that might exist between the two measures could be added to the bivariate model and result in separate effects on the sensitivity and specificity^54^. The standard output of this bivariate model includes the pooled logit sensitivity and specificity values with 95% confidence intervals. The summary receiver operating characteristic (sROC) curve was constructed and areas under the curve (AUC), which were calculated by use of bivariate models, showed the diagnostic performance of SW-MRI and standard MRI for the detection of calcifications. To assess whether significant heterogeneity, in the form of variance between the study estimates of sensitivity and specificity, was present, a chi-squared test (χ^2^) was performed. A covariate analysis was employed to further investigate sources of heterogeneity. To investigate publication bias, a regression test of asymmetry was performed^55^. A p-value of less than 0.05 was considered statistically significant. Meta-analytical data evaluation and creation of the graphs was performed using the freely available package ‘mada’ (version 0.5.7)^56^.

### Data availability

The datasets generated or analyzed in the course of the present meta-analysis can be requested from the corresponding author. All relevant data are within the paper and its Supporting Information files.

## Electronic supplementary material

Supplementary Information Tables S1 and S2
